# Gender Disparity in Academic Rank and Productivity Among Public Health Physician Faculty in North America

**DOI:** 10.7759/cureus.8553

**Published:** 2020-06-10

**Authors:** Donna Lee, Sabeena Jalal, Muazzam Nasrullah, Jeffrey Ding, Pina Sanelli, Faisal Khosa

**Affiliations:** 1 Family Medicine, Dalhousie University, Halifax, CAN; 2 Radiology, Vancouver General Hospital, Vancouver, CAN; 3 Emerging Infections, Centers for Disease Control and Prevention, Atlanta, USA; 4 Medicine, University of British Columbia, Vancouver, CAN; 5 Radiology, Northwell Health, Manhasset, USA

**Keywords:** usa, canada, gender disparity, public health, h-index, academic rank, research productivity

## Abstract

Background

The issue of gender disparity is particularly important in the domain of public health where the tone of its leadership is pivotal in bringing about impactful change to research, policies, and the wellbeing of our various populations. Our aim is to explore the gender disparity of author metrics and academic rankings of public health physician faculty through a cross-sectional study.

Methods

Data collection for this retrospective cross-sectional study took place during June and July of 2017. Public health and preventive medicine residency training programs in the United States and Canada were to compiled and all faculty members that met the inclusion criteria were recorded (n = 973). Variables of interest include gender, h-index, years of active research, and academic appointments. SCOPUS database (Elsevier, Amsterdam, the Netherlands) was used to generate author metrics, and all statistical tests were performed using Statistical Package for the Social Sciences (SPSS) software version 20 (IBM Corp., Armonk, NY).

Results

Overall, 31.14% (n = 303) of faculty members we studied were from Canada, and 68.86% (n = 670) were from the United States. In both countries, males made up the majority of all faculty members. Female faculty comprised most of the early career positions, and their proportions tapered off with higher academic rank, whereas male faculty trended in the opposite direction. Males generally were higher in all academic measures across all appointments.

Conclusions

Gender disparity exists within the North American public health and preventive medicine discipline. There are underlying factors preventing women from moving beyond early career positions or engaging in academic research.

## Introduction

The public health specialty implements strategies to improve the population wellbeing using modalities that include epidemiology, biostatistics, health policy, and international health [1]. Despite the broad scope of the public health specialty, women are still under-represented in higher-ranking positions, including at organizations such as the Centers for Disease Control (CDC), the World Health Organization (WHO), and the United Nations (UN) [2-4]. There still exists a substantial gap in the rate of promotion of women at the CDC compared to their male colleagues [2]. Women compromise 30% of members of the WHO Director-General’s office and about 25% of member state Chief Delegates to the World Health Assembly and Minsters of Health [3]. The same trend holds at the UN where much progress is needed to improve the status of women at senior levels [4]. These examples illustrate that the female leaders within public health are not at parity with their male colleagues.

In terms of academia, a recent paper by Schisterman looked at the gender disparity in relation to publication metrics within the epidemiology; they noted that female epidemiologists had fewer publications, while a greater number of them were in early career positions, compared to male counterparts [5]. Garcia-Calvente et al.’s 2015 study also looked at the gender inequalities in research in public health and epidemiology, but specifically focused on the population within Spain [6]. There is a paucity of research on gender disparity in academic rank and publication productivity among public health academics of North America. However, the gender disparity is well documented in medicine [7,8]. This disparity has been reported in medical specialties, professional societies, health administration bodies, and editorial boards [9-19]. This study aims to describe the gender disparity of author metrics and academic rankings of Canadian and American public health academics, where women, respectively, make up 51.7% and 33.8% of the discipline [20,21].

## Materials and methods

Data collection

This study was exempt from Institutional Review Board approval as no human subjects were involved and the data were retrieved from publicly available websites. Our methodology has been validated in several recent publications [11-16]. American and Canadian public health and preventive medicine residency training programs, between June and July 2017, were used to compile a list of all faculty members to which the inclusion and exclusion criteria were applied. Data analysis was conducted in the same year as the data were collected. The school listing for Canadian institutions was generated from the Canadian Resident Matching Service (CaRMS) website, which lists all residency training programs in Canada by specialty and by school [22]. We filtered for the schools offering “Public Health and Preventive Medicine” and “Preventive Medicine including Family Medicine” programs. The American residency programs were found through the American College of Preventive Medicine website, which provides a directory of all programs listed by state [23]. Significant overlap exists between the individual American residency programs and each school’s public health programs, so we also extended our search beyond the directory to include each school’s public health program (if they had one) to capture all appointed faculty. 

Faculty members were included if they met the criteria of being appointed an academic ranking of professor, associate professor, assistant professor, clinical professor, clinical associate professor, or clinical assistant professor. Faculty not having the above-mentioned academic ranks were excluded (i.e., adjunct, instructor, and retired or emeritus standings). All included faculty members possessed an MD, DO, or equivalent (such as Bachelor of Medicine-Bachelor of Surgery). Faculty who possessed leadership roles within the department, such as chairman, vice-chairman, program director, associate director, dean, associate/assistant directors, department head, division head, and chief were noted. Faculty who possessed a Master’s in Public Health (MPH) were also noted. Gender was identified using a combination of photos and names. If the gender was not initially apparent, we searched for gender information via biographical information, LinkedIn, Research Gate, or Healthgrades. Despite these efforts, if gender information was unavailable for any individual, the faculty member was excluded.

Upon generating a list of faculty members, Elsevier’s SCOPUS (Amsterdam, the Netherlands) was used to obtain author metrics such as the number of documents published, h-index, the total number of citations, number of citations by document, and the author’s earliest and latest years of publication. These variables provide an objective measure of publication productivity and the research impact of an author. The SCOPUS database was chosen as a tool for calculating h-index as it has the largest citation database of peer-reviewed research literature.

Efforts were made to carefully match and ensure each member was accurately represented, for example, individuals listed on SCOPUS who did not show the same affiliation with the school in question were cross-referenced using their publications listed on SCOPUS and those listed on their CV or website. For faculty who had a single publication, no further details were obtained other than the number of documents found on SCOPUS. When a faculty member did not have any listings or was not found, their metrics data was simply left blank. The entire data set was generated by the same individual to maintain consistency.

Statistical analysis

All statistical tests were performed using Statistical Package for the Social Sciences (SPSS) software version 20 (IBM Corp., Armonk, NY). The Mann-Whitney-U test (for two-category variables such as gender, MPH and country) and Kruskal-Wallis (for ordinal variables such as academic rank) were used to compare our continuous variables. The chi-square test was used to test the association between categorical variables. A two-tailed test with p < 0.05 was considered statistically significant.

The data were tested for normality. Log transformation was done for the continuous variables, which were skewed in distribution. At the univariate level, we chose the p-value of 0.25 for the cut-off. Each variable was regressed independently with h-index, their assumptions were checked, and their significance was reported. The dependent variable was h-Index and our main exposure of interest was gender. A step forward approach was used to build the model. A p-value of 0.05 was used as a cut-off to build the model with h-Index as the outcome. Co-variates were added one by one and tested for significance.

Variables that were significant on univariate regression were gender (beta coefficient: 12.00, p < 0.0001), publications (beta coefficient: 0.140, p < 0.0001), citations (beta coefficient: 0.0016, p < 0.0001), years of active research (beta coefficient: 1.032, p < 0.0001), academic ranks (clinical faculty: beta coefficient: 1.99, p = 0.481, < 0.0001 & professor: beta coefficient: 32.57, p < 0.0001), leadership ranks (beta coefficient: 5.06, p = 0.094), and MPH degree (beta coefficient: -2.35, p = 0.207). They were selected for inclusion into multivariable linear regression analysis. We checked for multi-collinearity between independent variables. 

Cramer’s V test was used for one nominal and one ordinal variable, while the Spearman test was used for one continuous variable and one ordinal variable. A correlation of > 0.8 was treated as the presence of multi-collinearity. There was no multi-collinearity seen. Leadership ranks were brought forward in the multivariable model but were again dropped from the model (p = 0.216). The multivariable analysis supported the inclusion of gender, citations, publications, academic rank, and years of research in the preliminary model. In order to build the final model, the final step was to check for interaction. Interaction terms were created between each of the main effects in the model. There was significant interaction between academic ranks and publications (associate professors: p = 0.03; assistant professors: p = 0.06; and professors: p = 0.0001). Academic ranks were significant (p = 0.03 and 0.001, respectively) and were included in the model.

The Final Model

 y(x) = β0 + β1 (Gender) + β2 (Publications) + β3 (Citations) + β41 (Academic Rank - Clinical Faculty) + β42 (Academic Rank- Associate Professor) + β43 (Academic Rank- Professor) + β5 (Years of research) + β61 (Academic Rank Clinical Faculty * Publications) + β62 (Academic Rank Associate Professor * Publications) + β63 (Academic Rank Professor * Publications)

This prediction equation accounted for major variability in the model as adjusted R square = 0.9201, F test = 826, p-value was < 0.001. The remaining variability in the model may have been explained by variables such as full-time versus part-time employment, years of employment, and contract versus tenure positions. However, this was beyond the scope of our paper, as we used the data that was available on the internet.

The odds ratio was calculated using binary logistic regression. Gender was the outcome variable and a model was built using a step forward technique. Female faculty in the field of Public Health had 1.22 times the odds of having a higher h-Index than male faculty, if all other variables are kept constant.

## Results

Faculty distribution by geography

**Figure 1:**
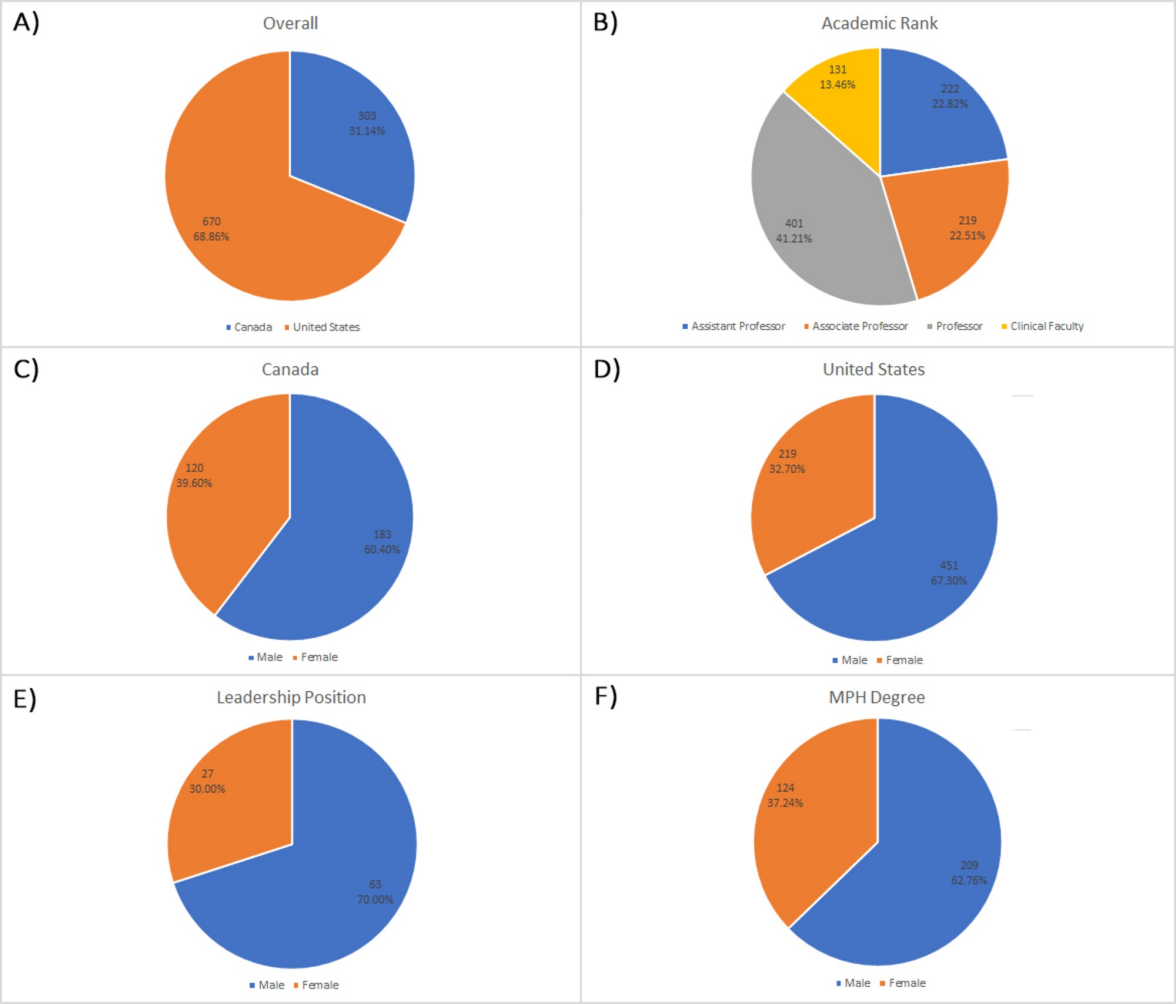
Pie charts of the various distributions of academic public health faculty members Panel A: Overall per cent composition of faculty members from Canada versus the United States. Panel B: Distribution of faculty members by academic rank. Panel C: Gender distribution of faculty members in Canada. Panel D: Gender distribution of faculty members in the United States. Panel E: Gender distribution of faculty members with a leadership position. Panel F: Gender distribution of faculty members with an MPH degree. Abbreviation: MPH, Master of Public Health

**Figure 2:**
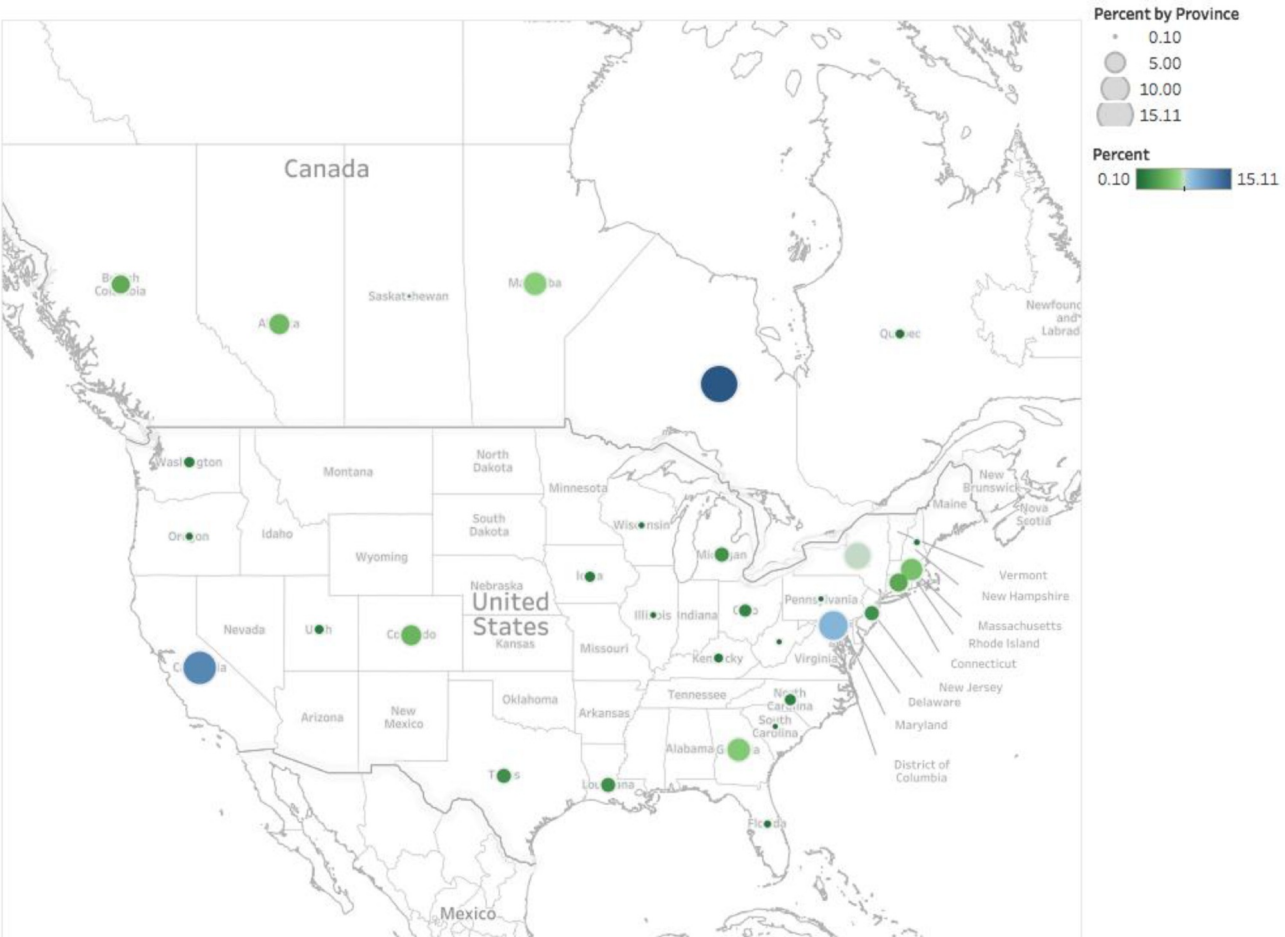
Percentage geographic distribution by province and state of public health physician faculty across Canada and the United States

There was a total of 973 faculty members, with 303 (31.14%) from Canada and 670 (68.86%) from the United States (Figure 1A). The overall distribution of physician faculty members across Canada and the United States is displayed in Figure 2. In Canada, the province or territory with the highest percentage of overall faculty was Ontario, and the lowest percentage was Saskatchewan (Table 1). The eastern provinces of Canada plus many of the Western and Midwestern states of the United States did not have residency programs and/or faculty present (Figure 2, Table 1). Males generally outnumbered females across the majority of provinces and states (31 total provinces and states), with the exception of Quebec, Kentucky, New Hampshire, South Carolina, and Texas.

**Table 1:** Geographic distribution of public health physician faculty across North America and according to province and state, stratified by gender and faculty appointment Male faculty comprise the majority in all provinces and states, except for Quebec, Kentucky, New Hampshire, and South Carolina. Provinces or states not listed did not have residency programs and/or faculty present. Abbreviation: USA, United States of America

Country	Province/State	Total	% Total	Female	% Female	Male	% Male	Professor	Associate Professor	Assistant Professor	Clinical Faculty
Canada	Alberta	48	4.93%	18	37.50%	30	62.50%	22	18	2	6
Canada	British Columbia	39	4.01%	14	35.90%	25	64.10%	6	1	1	31
Canada	Manitoba	60	6.17%	24	40.00%	36	60.00%	12	17	31	0
Canada	Ontario	147	15.11%	57	38.78%	90	61.22%	53	44	50	0
Canada	Quebec	8	0.82%	7	87.50%	1	12.50%	2	3	3	0
Canada	Saskatchewan	1	0.10%	0	0.00%	1	100.00%	0	1	0	0
USA	California	118	12.13%	29	24.58%	89	75.42%	36	14	25	43
USA	Colorado	45	4.62%	21	46.67%	24	53.33%	22	10	10	3
USA	Connecticut	37	3.80%	10	27.03%	27	72.97%	25	7	4	1
USA	Florida	5	0.51%	1	20.00%	4	80.00%	1	3	1	0
USA	Georgia	57	5.86%	18	31.58%	39	68.42%	31	15	11	0
USA	Illinois	4	0.41%	2	50.00%	2	50.00%	3	0	1	0
USA	Iowa	11	1.13%	3	27.27%	8	72.73%	5	0	1	5
USA	Kentucky	8	0.82%	5	62.50%	3	37.50%	1	2	5	0
USA	Louisiana	23	2.36%	9	39.13%	14	60.87%	8	7	4	4
USA	Maryland	92	9.46%	29	31.52%	63	68.48%	55	19	17	1
USA	Massachusetts	52	5.34%	12	23.08%	40	76.92%	36	7	8	1
USA	Michigan	24	2.47%	7	29.17%	17	70.83%	20	2	1	1
USA	New Hampshire	4	0.41%	4	100.00%	0	0.00%	0	1	3	0
USA	New Jersey	23	2.36%	7	30.43%	16	69.57%	11	6	6	0
USA	New York	73	7.50%	30	41.10%	43	58.90%	21	18	15	19
USA	North Carolina	14	1.44%	4	28.57%	10	71.43%	7	3	4	0
USA	Ohio	17	1.75%	6	35.29%	11	64.71%	3	8	6	0
USA	Oregon	6	0.62%	0	0.00%	6	100.00%	2	1	3	0
USA	Pennsylvania	3	0.31%	1	33.33%	2	66.67%	1	1	1	0
USA	South Carolina	3	0.31%	2	66.67%	1	33.33%	0	0	3	0
USA	Texas	23	2.36%	12	52.17%	11	47.83%	11	6	6	0
USA	Utah	9	0.92%	1	11.11%	8	88.89%	3	0	0	6
USA	Washington	12	1.23%	4	33.33%	8	66.67%	1	2	0	9
USA	West Virginia	3	0.31%	1	33.33%	2	66.67%	1	2	0	0
USA	Wisconsin	4	0.41%	1	25.00%	3	75.00%	2	1	0	1
Total		973		339	34.84%	634	65.16%	401	219	222	131

Faculty distribution by ranking

Overall, of the total faculty members, there were 222 (22.82%) assistant professors, 219 (22.51%) associate professors, 401 (41.21%) professors, and 131 (13.46%) were clinical faculty with cross-appointments in various public health schools (Figure 1B). The percentage distribution of faculty positions was similar between Canada and the United States, where most faculty held a professor title and fewer held titles as clinical faculty. As can be observed in Table 2, there were statistically significant differences in the gender distributions within the academic ranks of professor (p < 0.0001), associate professor (p < 0.0001), and assistant professor (p < 0.0001). No statistically significant difference in the gender distribution was noted for individuals with the appointment of clinical faculty (p = 0.481). 

**Table 2:** Academic productivity metrics by gender, country, and academic position for public health physician faculty in North America P <0.05 indicates a statistically significant difference in the gender distribution of the respective academic rank. Abbreviation: USA, United States of America

	Nation	Total	% by Gender	Leadership Title	% by Gender	MPH Degree	% by Gender	H-index (Median, Range) by Gender	Publications (Median, Range) by Gender	Citations (Median, Range) by Gender	Years of Research (Median, Range)	p-value
Professors												< 0.0001
Male	USA	314	78.30%	42	79.66%	85	81.51%	38.5 (1-228)	182 (1-1733)	6506 (0-191025)	29 (0-68)	
	Canada	238		5		12						
Female	USA	68	21.70%	9	20.34%	21	18.49%	32.5 (3-137)	127.5 (10-1022)	3881.5 (91-83327)	27 (14-65)	
	Canada	19		3		1						
Associate Professor												< 0.0001
Male	USA	84	57.53%	4	40.00%	32	56.72%	18 (1-60)	50 (1-319)	990.5 (0-13597)	17 (1-55)	
	Canada	42		0		6						
Female	USA	51	42.47%	5	60.00%	21	43.28%	16 (1-48)	51 (1-248)	846 (4-11902)	17 (3-44)	
	Canada	42		1		8						
Assistant Professor												< 0.0001
Male	USA	64	45.95%	5	54.55%	29	29.89%	10 (1-44)	21.5 (1-192)	400.5 (0-10500)	13.5 (2-42)	
	Canada	38		1		7						
Female	USA	71	54.05%	5	45.45%	42	58.62%	7 (1-36)	13 (1-159)	251 (2-4239)	12 (1-62)	
	Canada	49		0		9						
Clinical Faculty												0.481
Male	USA	65	70.23%	6	60.00%	32	46.67%	8 (1-94)	16 (1-425)	266.5 (6-2779)	17 (3-32)	
	Canada	27		0		6						
Female	USA	29	29.77%	4	40.00%	18	36.67%	6 (1-25)	11 (1-100)	275 (3-45225)	23.5 (1-43)	
	Canada	10		0		4						
p-value				0.216		0.207		< 0.0001	< 0.0001	< 0.0001	< 0.0001	

Faculty distribution by gender

In both countries, males made up the majority of all faculty members (Table 1). In Canada, there were 120 (39.60%) female faculty, and 183 (60.40%) male faculty (Figure 1C). In the United States, there were 219 (32.70%) female faculty and 451 (67.30%) male faculty (Figure 1D).

**Figure 3:**
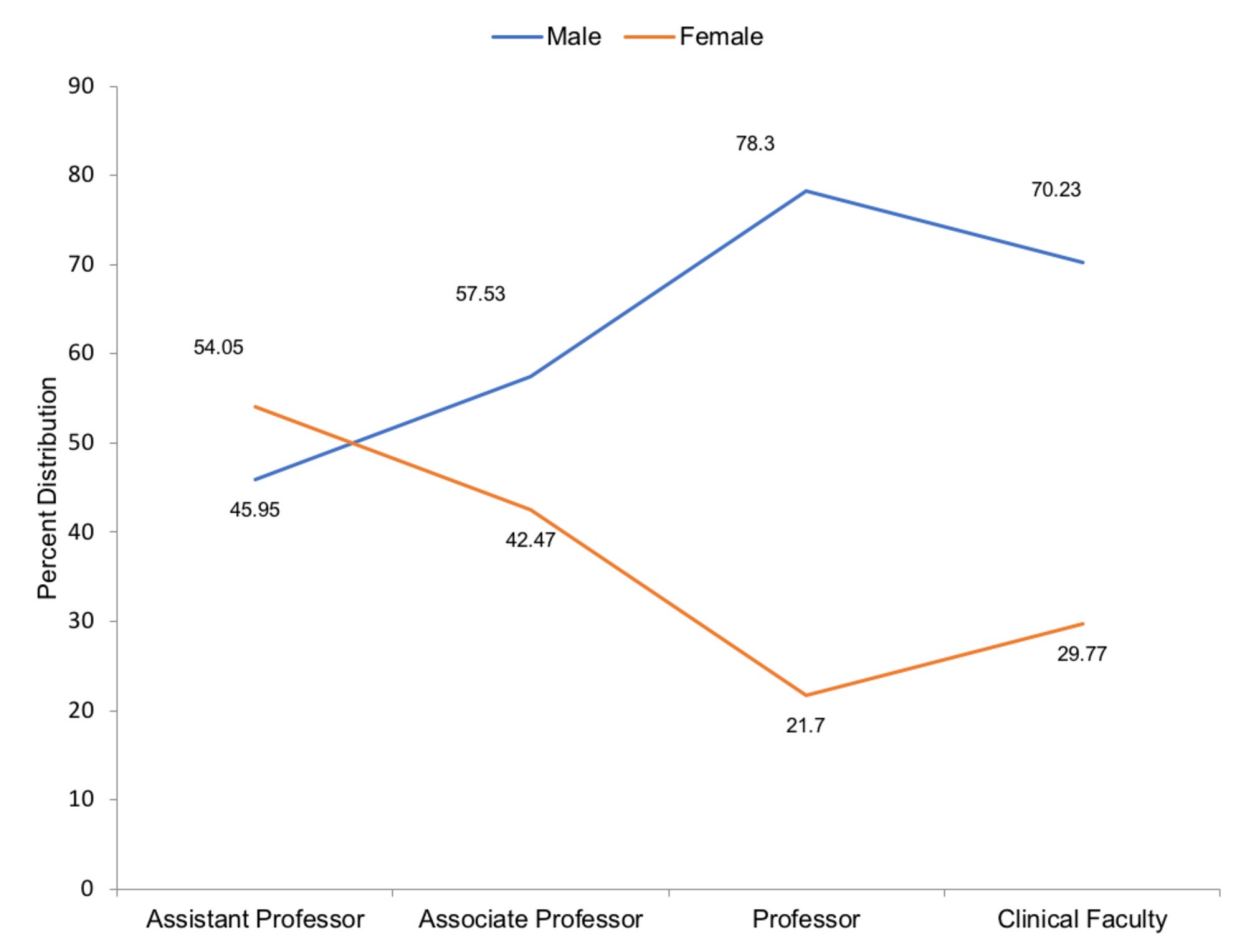
Gender distribution by percent within each faculty appointment across all public health physician faculty members

Females were only in greater number compared to males within the assistant professor ranking. Their numbers drop, whereas male numbers rise as one progresses through to professor ranking as seen in Figure 3. The majority of male faculty members held the professor title, whereas the majority of females held the assistant professor title. Despite having more positions with ascending academic rank, females were fewer in number at higher positions as they mainly held early career positions compared to males.

Faculty distribution by leadership ranking and MPH degree

There were 90 faculty members identified in leadership positions. This included roles such as chairman, vice-chairman, program director, associate director, dean, associate/assistant directors, department head, division head, and chief. Of this total, 27 (30%) were female faculty and 63 (70%) were male faculty (Figure 1E). There was not a statistically significant difference in gender distribution in terms of leadership position (p = 0.094).

A total of 333 faculty members held an MPH degree, where 124 (37.24%) were held by females and 209 (62.76%) were held by males (Figure 1F, Table 2). There was not a statistically significant difference in gender distribution within this parameter (p = 0.207).

Publication productivity

We looked at several parameters to gauge academic productivity. Applying the Mann-Whitney U-test, we noted that there was a statistically significant difference between male and female h-index (Z = -6.682; p = 0.0001), number of publications (Z = -7.523, p <0.0001), number of citations (Z = -6.472, p < 0.0001), and number of years of active research (Z = - 6.696, p < 0.0001). We can generally observe that the male faculty had greater h-index, publications, and citations than the female faculty across all faculty positions, with the exception of clinical faculty (Table 2).

## Discussion

In echoing similar sentiments expressed for other specialties in medicine, our study shows that women are significantly under-represented in academic public health by way of numbers, academic productivity, and ascension into higher academic rankings in Canada and the United States. Interestingly, our study found that the genders did not differ in terms of education by the measure of holding an MPH and departmental leadership roles. This may suggest that opportunities appear available to the fewer women in public health, but the majority of women do not go further in their careers for reasons other than differences in educational qualification or possible opportunity to take on leadership roles [24,25].

Implications for practice and/or policy

Factors that may be preventing women from progressing further in their public health careers may relate to several levels of influence, specifically at the individual, interpersonal, institutional, academic community, and policy levels [26]. Carr et al. outline that despite greater numbers of women entering medicine, this has not resulted in more women achieving senior positions. Through their study, Carr et al. found that many US medical schools did not have programs in place that supported gender equity among its medical faculty [24]. To elaborate, programs were not in place for recruiting, promoting, or retaining women and the primary reason given from their survey showed that there was a perceived lack of need for such programs - a belief that appears to be unsupported by statistics on gender differences in senior positions amongst academic medical faculty. This mirrors one of the several challenges expressed by the UN’s report on improvement in the status of women in the UN system, where a lack of special measures for gender equality and insufficient outreach was a barrier to gender balance [4]. The findings in our study undoubtedly support the need to explore the policies, or lack of policies, to promote the advancement of women’s careers in public health programs and warrant further study in this regard.

Furthermore, on the issue of policies and practices, not only is it important to have explicit policies in place for promoting gender parity but also such policies must provide flexibility. There are new generational expectations and perspectives with achieving work-life balance and gender roles make it especially challenging for women to achieve such balance [24]. Further, on the gender roles, hours of market work were indistinguishable between male and female physicians when unmarried and with no children; however, this changes with marriage and children [25]. Men have higher market hours and their hours were unchanged or increased with parenthood, whereas females have lower market hours and much lower hours as a parent. Women tended to feel distressed, guilty, or judged when faced with competing responsibilities between career and motherhood [25]. Certainly, motherhood is a large factor affecting the trajectory that a woman’s career may take, but measures should be in place to implement flexible work arrangements that facilitate these competing demands to be met if desired.

Mentorship programs throughout a physician’s career are important. Receiving mentorship is critical to the female leaders who catalyze positive community change in public health [27]. The presence of female mentors with families serves as a significant role model for both men and women, who provide inspiration and advice based on personal experiences; the lack of a mentor in this position played a large role for physicians leaving academic medicine [27]. Despite having male mentors available, females still felt disadvantaged because they couldn't truly relate or provide appropriate advice [27]. In regards to public health, it is challenging when women are still the minority, and thus, fewer female mentors are available. However, this is an opportunity for us to emphasize the importance of encouraging and fostering the careers of women in public health. Encouraging females in senior positions to mentor women who are early in their careers is imperative.

Limitations

Our study was subject to a few limitations. The information regarding academic ranks that we collected was in large part institution-dependent, which may not be up to date at the time of data collection. Some institutions rely on faculty members to submit their curriculum vitae or profile information, which is affected by inconsistencies and compliance issues.

Faculty members who have changed either their first or surname may not have been correctly identified in the SCOPUS database. This could have resulted in individuals being assigned the incorrect author metrics, or if they were missing altogether in the SCOPUS database, the faculty member would have been excluded from the analysis.

A measure of education or credential equivalencies between genders is not solely measured by possession of an MPH, so further elucidation of educational attainments may be considered.

The demographics, populations, and the number of institutions available in each country differ and this may influence the number of and distribution of professionals entering the public health discipline. In addition, the cultural attitudes and expectations towards women, and their roles within educational institutions and research, may shape the types and numbers of opportunities available. Finally, the educational systems and programs available to pursue public health may also differ between Canada and the United States. It is uncertain if the results in this study can be generalized to other regions of the world.

A limitation due to the study design is that it cannot be determined if academic productivity is a result of academic standing and/or if academic productivity leads to higher ranks. We cannot be sure if females are simply choosing not to enter high academic rankings or if they are limited by their lack of academic productivity.

## Conclusions

Gender disparity continues to exist within the public health and preventive medicine discipline. There are underlying factors preventing women from moving beyond early career positions and/or engaging in academic research. Future directions include exploring the specific programs and policies in place to facilitate the recruitment, promotion, and retainment of female faculty members. Furthermore, there is a need to investigate whether such policies are enforced, to ensure that work arrangements are accommodating and flexible, and to ascertain the presence and availability of female mentors.

## References

[1] (2019). Objectives of training in the specialty of public health and preventive medicine. http://www.royalcollege.ca/rcsite/documents/ibd/public-health-preventive-medicine-otr-e.pdf.

[2] Chen Z, Roy K, Gotway-Crawford C (2010). Examining the role of gender in career advancement at the Centers for Disease Control and Prevention. Am J Public Health.

[3] Dhatt R, Kickbusch I, Thompson K (2017). Act now: a call to action for gender equality in global health. Lancet.

[4] (2019). Improvement in the status of women in the United Nations system: report of the secretary-general. https://www.unwomen.org/en/digital-library/publications/2014/8/improvement-of-the-status-of-women-in-the-un-system-2014.

[5] Schisterman EF, Swanson CW, Lu YL, Mumford SL (2017). The changing face of epidemiology: gender disparities in citations?. Epidemiology.

[6] García-Calvente M, Ruiz-Cantero MT, Del Río-Lozano M, Borrell C, López-Sancho MP (2015). Gender inequalities in research in public health and epidemiology in Spain (2007-2014). Gac Sanit.

[7] Abdellatif W, Ding J, Jalal S (2019). Leadership gender disparity within research-intensive medical schools: a transcontinental thematic analysis. J Contin Educ Health Prof.

[8] Moghimi S, Khurshid K, Jalal S (2019). Gender differences in leadership positions among academic nuclear medicine specialists in Canada and the United States. Am J Roentgenol.

[9] Waseem Y, Mahmood S, Siddiqi R (2019). Gender differences amongst board members of endocrinology and diabetes societies. Endocrine.

[10] Odell T, Toor H, Takayanagi A (2019). Gender disparity in academic neurosurgery. Cureus.

[11] Abdellatif W, Shao M, Jalal S (2019). Novel geographic thematic study of the largest radiology societies globally: how is gender structure biased within editorial boards?. AJR Am J Roentgenol.

[12] Wu B, Bhulani N, Jalal S, Ding J, Khosa F (2019). Gender disparity in leadership positions of general surgical societies in North America, Europe, and Oceania. Cureus.

[13] Chen ST, Jalal S, Ahmadi M (2020). Influences for gender disparity in academic family medicine in North American medical schools. Cureus.

[14] Hafeez DM, Waqas A, Majeed S (2019). Gender distribution in psychiatry journals' editorial boards worldwide. Compr Psychiatry.

[15] Khan MS, Usman MS, Siddiqi TJ (2019). Women in leadership positions in academic cardiology: a study of program directors and division chiefs. J Women's Health.

[16] Abdellatif W, Ding J, Jalal S (2020). Lack of gender disparity among administrative leaders of Canadian health authorities [Epub ahead of print]. J Women's Health.

[17] Wang J, Khurshid K, Jalal S (2019). Influence of academic productivity on gender disparity in academic interventional radiology. Am J Roentgenol.

[18] Shah A, Jalal S, Khosa F (2018). Influences for gender disparity in dermatology in North America. Int J Dermatol.

[19] Qamar SR, Khurshid K, Jalal S (2020). Gender disparity among leaders of Canadian academic radiology departments. Am J Roentgenol.

[20] (2019). Number and percent distribution of physicians by specialty and sex. https://www.cma.ca/sites/default/files/2019-03/2018-06-spec-sex.pdf.

[21] (2019). Active physicians by sex and specialty. https://www.aamc.org/data-reports/workforce/interactive-data/active-physicians-sex-and-specialty-2017.

[22] (2017). Program descriptions - first iteration. https://www.carms.ca/en/residency/r-1/program-descriptions/.

[23] (2017). Preventive medicine residency programs. https://www.acpm.org/education-events/residency-program/.

[24] Carr PL, Gunn C, Raj A, Kaplan S, Freund KM (2017). Recruitment, promotion, and retention of women in academic medicine: how institutions are addressing gender disparities. Women's Health Issues.

[25] Strong EA, De Castro R, Sambuco D (2013). Work-life balance in academic medicine: narratives of physician-researchers and their mentors. J Gen Intern Med.

[26] Wang C, Sweetman A (2013). Gender, family status and physician labour supply. Soc Sci Med.

[27] Folta SC, Seguin RA, Ackerman J, Nelson ME (2012). A qualitative study of leadership characteristics among women who catalyze positive community change. BMC Public Health.

